# Anhedonia to Gentle Touch in Fibromyalgia: Normal Sensory Processing but Abnormal Evaluation

**DOI:** 10.3390/brainsci10050306

**Published:** 2020-05-18

**Authors:** Rebecca Boehme, Helene van Ettinger-Veenstra, Håkan Olausson, Björn Gerdle, Saad S. Nagi

**Affiliations:** 1 Center for Social and Affective Neuroscience, Linköping University, 58185 Linköping, Sweden; hakan.olausson@liu.se (H.O.); saad.nagi@liu.se (S.S.N.); 2 Center for Medical Image Science and Visualization (CMIV), 58185 Linköping, Sweden; helenevanettinger@gmail.com (H.v.E.-V.); bjorn.gerdle@liu.se (B.G.); 3 Pain and Rehabilitation Centre, and Department of Health, Medicine and Caring Sciences, Linköping University, 58185 Linköping, Sweden; 4 Department of Clinical Neurophysiology, Linköping University, 58185 Linköping, Sweden

**Keywords:** touch, pain, C-tactile afferents, fibromyalgia, anhedonia, fMRI, posterior insula

## Abstract

Social touch is important for interpersonal interaction. Gentle touch and slow brushing are typically perceived as pleasant, the degree of pleasantness is linked to the activity of the C-tactile (CT) fibers, a class of unmyelinated nerves in the skin. The inability to experience pleasure in general is called anhedonia, a common phenomenon in the chronic pain condition fibromyalgia. Here, we studied the perception and cortical processing of gentle touch in a well-characterized cohort of fibromyalgia. Patients and controls participated in functional brain imaging while receiving tactile stimuli (brushing) on the forearm. They were asked to provide ratings of pleasantness of the tactile stimulus and ongoing pain. We found high distress, pain catastrophizing, and insomnia, and a low perceived state of health in fibromyalgia. Further, patients rated both slow (CT-optimal) and fast (CT-suboptimal) brushing as less pleasant than healthy participants. While there was no difference in brain activity during touch, patients showed deactivation in the right posterior insula (contralateral to the stimulated arm) during pleasantness rating and activation during pain rating. The opposite pattern was observed in healthy participants. Voxel-based morphometry analysis revealed reduced grey matter density in patients, in the bilateral hippocampus and anterior insula. Our results suggest anhedonia to gentle touch in fibromyalgia with intact early-stage sensory processing but dysfunctional evaluative processing. These findings contribute to our understanding of the mechanisms underlying anhedonia in fibromyalgia.

## 1. Introduction

Fibromyalgia (FM) is a common, debilitating chronic pain condition. According to the American College of Rheumatology 1990 Criteria for the Classification of Fibromyalgia [1], the cardinal features comprise widespread pain and hypersensitivity, i.e., pain evoked by non-painful stimuli and exaggerated pain to painful stimuli. FM includes a myriad of other symptoms, including fatigue, sleep, and affective disturbances (e.g., anxiety and depression). The prevalence of FM is higher among females [2].

Social touch is important for human behavior and communication between individuals [3]. Pleasantness (or pleasure) associated with skin-to-skin contact is linked to a class of nerves in the skin, called C-tactile (CT) fibers. This is of considerable interest for questions about physical and social well-being and the interoceptive system [3]. CTs exhibit a unique ‘inverted U-shaped’ response pattern to brushing velocities, with slow brushing (1–10 cm/s, ‘CT-optimal’) producing a robust discharge—a stimulus that is perceived as pleasant and preferred by participants over fast brushing [4]. CTs have also been implicated in pain processing either directly or indirectly, as an allodynic substrate itself or through malfunctioning of this network [5,6].

While unmyelinated nociceptors have been the focus of earlier studies on fibromyalgia [7], less is known about the role of their low-threshold counterparts, the CTs. We have previously shown a blunted affective distinction between slow CT-optimal and fast CT-suboptimal brushing in FM patients, compared to healthy participants [8], suggesting a reduced CT input or processing in FM.

CT-optimal touch produces robust activation of the posterior insula with a somatotopic organization similar to that reported for cutaneous and muscle pain in healthy participants [9,10]. FM patients show higher activity in the insular cortex in response to painful stimuli (5), and have higher levels of glutamate in the posterior insula, an excitatory neurotransmitter associated with clinical pain and mechanical hypersensitivity in FM [11].

In the current study, we hypothesized to find differences in neural processing in the insular cortex, in response to CT-optimal touch in FM. In a group of well-characterized FM patients, we used fMRI to examine the cortical responses to slow and fast brush stroking and compared with matched healthy controls. In parallel, ratings for touch pleasantness and ongoing pain were collected. Grey matter density was also measured.

## 2. Materials and Methods

### 2.1. Participants

Female patients with a clinical diagnosis of FM, ranging between 25–55 years old, were recruited through the Pain and Rehabilitation Center at the Linköping University Hospital. These patients were recruited as part of a broader characterization of FM. Age- and gender-matched healthy controls (HC) were recruited through advertisements at the Linköping University and the University Hospital, and in the local news media. The Linköping Regional Ethics Review Board approved the study (Dnr 2016/471-32) and written informed consent was obtained after participants had read the complete study description, in accordance with the Declaration of Helsinki.

On the first visit, all participants underwent a clinical examination. To confirm the patients’ eligibility, the eighteen tender points according to the FM classification criteria of the American College of Rheumatology [1] were clinically examined by senior consultants in rheumatology or pain medicine. This was performed in both patients and HC. Revised criteria for FM were recently presented [12,13], but these were not available in the finished form when the current study was designed and ethical approval was sought. The clinical examination also included registration of systolic and diastolic blood pressures. Weight (kg) and height (m) were also registered; body mass index (BMI; kg/m^2^) was calculated as weight/height^2^. At the time-point of the clinical examination, the subjects answered a health questionnaire covering demographic data as well as pain and psychological characteristics.

Exclusion criteria were MRI-incompatibility (claustrophobia or metal in the body), pregnancy, difficulty understanding Swedish, metabolic disease, neurological disease or severe psychiatric conditions, malignancy, rheumatoid arthritis, unregulated thyroid disease, cardiovascular disease, or lung disease. Another exclusion criterion was the inability to refrain from analgesics, including NSAIDs and sleep medication, for 48 h prior to the fMRI visit (i.e., a 48-h pharmacological washout period). Participants in the HC group reported having no current pain.

Functional imaging data of good quality were obtained for 31 patients (mean age, 39.0 ± 11.4 years) and 29 matched controls (mean age 42.7 ± 10.1 years).

### 2.2. Background Data

Age and gender were registered. FM patients also reported the duration of their condition in years.

#### 2.2.1. Pain Intensity Aspects

A numeric rating scale (NRS) with anchor points 0 (denoting no pain) and 10 (denoting the worst imaginable pain) was used to capture the current pain (denoted by ‘Pain Intensity Current’) and the average pain intensity for the previous four weeks (denoted by ‘Pain Intensity 4w’).

#### 2.2.2. Psychological Distress

The Hospital Anxiety and Depression Scale (HADS) was used to measure symptoms of anxiety and depression. The validated Swedish translation of HADS was chosen to reflect aspects of psychological distress [14,15] and had good psychometric characteristics [15,16]. HADS contains seven items in each of the depression and anxiety subscales (HAD-Depression and HAD-Anxiety). Both subscale scores ranged from 0–21. A score of 7 or less on each subscale was considered normal, a score of 8 to 10 indicated a possible abnormality, and a score of 11 or more indicated a definite abnormality [15]. In the present study, a score ≥ 11 was considered as having severe anxiety and depression symptoms.

#### 2.2.3. Sleeping Problems

The Insomnia Severity Index (ISI) was used to quantify perceived insomnia severity. ISI captures the severity and impact of insomnia symptoms with good validity and internal consistency [17,18]. The seven items of ISI were rated on a five-point Likert scale (0–4). The scores of each item were added to calculate the total score of ISI (max = 28). The score could be divided into four categories–no insomnia (0–7), sub-threshold insomnia (8–14), moderate insomnia (15–21), and severe insomnia (22–28).

#### 2.2.4. Pain Catastrophizing Scale (PCS)

PCS measures three dimensions of catastrophizing—rumination, magnification, and helplessness [19,20]—based on 13 items with anchor points ranging from 0 (not at all) to 4 (all the time). The current study used the total PCS (PCS-total); 52 was the maximum score according to the original scale and a high score represented a worse outcome. This instrument had good internal consistency, test-retest reliability, and validity [21].

#### 2.2.5. Impact of the Pain Aspects

An NRS with anchor points 0 (denoted not at all) and 10 (denoted impossible to perform these activities) was used to capture to what extent the pain hindered daily activities (pain hindrance activities of daily living (ADL)), taking part in leisure activities including social and family activities (pain hindrance leisure), and working, including studies or homework (pain hindrance work). These items were only answered by the FM group.

#### 2.2.6. The European Quality of Life Instrument (EQ-5D)

EQ-5D captured a patient’s perceived state of health [22,23,24]. The first part of the instrument captured five dimensions—mobility, self-care, usual activities, pain/discomfort, and anxiety/depression. In the present study, we used the second part of the instrument and measured the current day’s health on a 100-point scale, a thermometer-like scale (EQ-VAS) with defined endpoints (high values indicated good health and low values indicated bad health).

#### 2.2.7. Pharmacological Treatments

Participants were asked to report their ongoing pharmacological treatments. Note that those included in the study were asked to refrain from analgesic use, including NSAIDs and sleep medication, for 48 h prior to the fMRI visit.

### 2.3. Stimuli and Procedures

#### 2.3.1. Task

Before entering the MRI scanner, participants were instructed that they will be brushed on the forearm and were shown the rating scales. They were also brushed once on the forearm. During MRI, participants were brushed on the left forearm at two different velocities—3 cm/s (CT-optimal) and 30 cm/s (CT-suboptimal). The brusher, who was trained in the delivery of these stimuli, stood next to the scanner bore and received auditory cues over headphones. A 9-cm long line with 3-cm increments, was marked on the left forearm to aid the brusher to maintain the correct speed. The task consisted of three runs of 6 min each. In between runs, the participants were asked if they were alright in the scanner and okay to continue with the next run. In total, there were 15 repetitions of slow and fast brushing blocks, each lasting 12 s. The intertrial interval (ITI) between blocks was 10 to 12 s. The order of brushing velocities was randomized. During ITIs, participants looked at a fixation cross. Three times per condition (slow or fast), following a 10 to 12-s ITI, a question appeared on the screen asking the participant to rate the pleasantness of the brushing (“How pleasant is the stimulus?”) and if they felt any pain (“Do you feel any pain?”). We formulated the latter question in this specific way because we assumed it might be difficult for the FM group to disentangle ongoing pain and specific pain related to the stimulus. This rating was therefore to be understood as rating of any ongoing pain. Using a button box in their right hand, participants moved a cursor on a visual analog scale (VAS) between the endpoints “unpleasant–pleasant” or “no pain–intense pain” (in Swedish). During analysis, the cursor positions were converted to their numerical values, ranging from −10 to +10 for pleasantness and 0 to 20 for pain. The pain ratings were not specifically related to brush-evoked sensation but to the subjects’ general pain level.

#### 2.3.2. MRI

Participants laid comfortably in a 3.0 Tesla Siemens scanner (Prisma, Siemens, Erlangen, Germany). Their left arm was placed on their belly and propped up by pillows. A 12-channel head coil was used to acquire 295 T2-weighted echo-planar images (EPI) per run, containing 48 multiband slices (TR = 1030 ms, TE = 30 ms, slice thickness = 3 mm, matrix size = 64 × 64, field of view = 488 × 488 mm, in-plane voxel resolution = 3 mm², flip angle = 63°). T1-weighted anatomical images were also acquired.

#### 2.3.3. Analysis

Behavioral data were analyzed using SPSS (IBM Corp., Armonk, NY, USA). Ratings for slow and fast blocks were averaged. Ratings of the patient group were normally distributed; ratings of the control group were not due to a strong ceiling effect. Ratings were compared between conditions using paired t-test (FM) and Wilcoxon test (HC), and between groups, using Mann–Whitney U test. *p*-values < 0.05 were considered significant.

Functional MRI data were analyzed using statistical parametric mapping (SPM12, Wellcome Department of Imaging Neuroscience, London, UK; http://www.fil.ion.ucl.ac.uk/spm) in Matlab R2016a (The MathWorks, Natick, MA, USA). The following steps were performed—motion correction, co-registration of the mean EPI and the anatomical image, spatial normalization to the MNI T1 template, and segmentation of the T1 image, using the unified segmentation approach [25]. Normalization parameters were applied to all EPIs. All images were spatially smoothed with an isotropic Gaussian kernel of 6 mm full width, at half maximum. For statistical analysis of the BOLD response, the general linear model approach was used as implemented in SPM12. Using a block-design, the conditions (slow and fast) and the rating phase were convolved with the hemodynamic response function. Motion parameters were added as regressors of no interest. Family-wise-error (FWE) correction on the voxel level was used to correct for multiple comparisons on the whole-brain level and using small volume correction based on our a priori regions of interest (ROI). Based on previous studies [10,26], we were specifically interested in posterior insula activations, therefore, a posterior insula mask was used for small volume corrections (SVC) [27].

#### 2.3.4. VBM

Voxel-based morphometry (VBM) was estimated using the VBM routine provided by the CAT12 toolbox (Gaser & Dahnke, Jena University Hospital, Departments of Psychiatry and Neurology) in SPM12. Total intracranial volume was included as a covariate. Groups were compared using a *t*-test at the whole brain level.

## 3. Results

### 3.1. Clinical Characteristics

The disease duration in the FM cohort was 4.4 years, on average, since diagnosis and as expected, they had a high number of tender points, i.e., at the group level, >16 out of 18. In HC, the tender points were scarcely found. FM patients had significantly higher blood pressure and BMI than HC. While HC reported no pain, both pain intensity measures (current and at 4 weeks) in FM patients were above 5 on the NRS. Distress, pain catastrophizing, and insomnia (HADS, PCS, and ISI) were significantly higher and overall health (EQ-VAS) was significantly lower in FM patients (Table 1).

Four FM patients had more severe symptoms (≥11) according to the HADS-Depression subscale, compared to none in the HC. Corresponding figures for the HADS-Anxiety were eight patients in FM and none in HC. At least moderate insomnia (≥15) was found in 14 FM patients and in one HC.

Three FM patients and 26 HC did not use any pharmacological drugs. Those using medication among the FM cohort were often on several substances (range 1–5), including paracetamol (*n* = 19), antidepressant medication (selective serotonin reuptake inhibitor and serotonin-norepinephrine reuptake inhibitor, *n* = 12), tricyclic antidepressants (*n* = 9), opioids (*n* = 7), vitamins (e.g., B12, *n* = 5), medication for high blood pressure (*n* = 3), proton-pump inhibitors (*n* = 3), gabapentin (*n* = 2), antihistamine (*n* = 2), and other medications (*n* = 9). HC reported sumatriptan (*n* = 1), tricyclic antidepressant (*n* = 1), methotrexate (*n* = 1), and metoprolol (*n* = 1). All participants refrained from analgesics including NSAIDs and sleep medication for 48 h prior to the fMRI visit (cf. Methods).

### 3.2. Behavior

**Figure 1:**
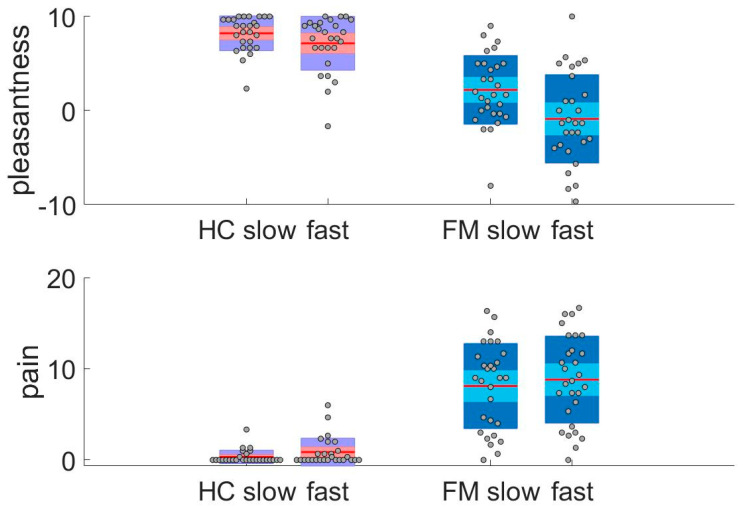
Ratings of touch pleasantness and pain during fMRI. Top: HC rated slow and fast brushing as similarly pleasant on a VAS (mean slow = 8.2 ± 1.9, mean fast = 7.2 ± 3, Z = –1.6, *p* < 0.103). FM rated slow brushing as significantly more pleasant than fast (mean slow = 2.1 ± 3.7, mean fast = –0.9 ± 4.7, *t* = 4.9, *p* < 0.001). Groups differed in their ratings of the different conditions (Mann–Whitney U test, pleasantness ratings: slow Z = –5.7, *p* < 0.001, fast Z = –5.4, *p* < 0.001). Bottom: HC rated less pain after slow than after fast brushing on a VAS (mean slow = 0.33 ± 0.04, mean fast = 0.86 ± 1.53, Z = –2.4, *p* < 0.017). FM rated less pain after slow than after fast brushing (mean slow = 8.3 ± 4.7, mean fast = 9 ± 4.9, *t* = –2.7, *p* < 0.011). The groups differed in their ratings of the different conditions (Mann–Whitney U test, pain ratings: slow Z = –6.2, *p* < 0.001, fast Z = –5.9, *p* < 0.001). Note that the pain ratings were not specifically related to brush-evoked sensation but to the subjects´ general pain level.

HC rated slow and fast brushing as similarly pleasant (Figure 1, top panel). Within the FM patient group, slow brushing was rated as pleasant, while fast brushing was rated as unpleasant. Affective ratings to slow and fast brushing were lower in FM patients than HC. Pain was rated very low in HC, as expected (Figure 1, bottom panel). Notwithstanding, HC reported less pain to slow than to fast brushing. In FM, pain was also rated somewhat lower after slow than after fast brushing. As described in the Methods, the pain ratings were not specifically related to brush-evoked sensation but to the subjects’ general pain level. In summary, the HC and FM groups differed in their ratings of touch pleasantness and ongoing pain. FM rated both slow and fast brushing as less pleasant than HC. In addition, pain ratings in FM were higher than HC.

### 3.3. Functional Imaging

**Figure 2:**
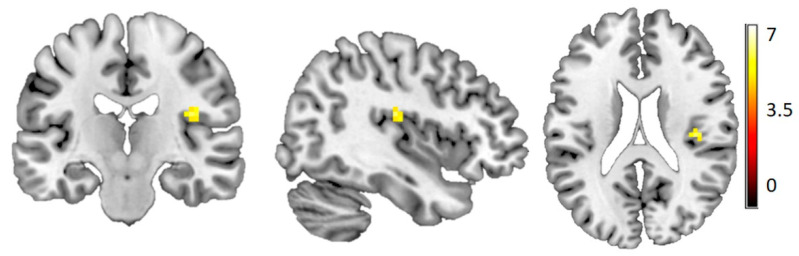
More activation in the posterior insula during slow brushing compared to fast brushing. In all participants, [39–1920] *t* = 6.25, Z = 5.79, FWE corrected at the whole-brain level *p* < 0.05, color scale depicts *t*-values for the contrast slow > fast.

Across all participants, we found a significantly stronger activation for slow compared to fast brushing in the right posterior insula (contralateral to the stimulated forearm) (Figure 2). There was no main effect of group and no difference between groups for separate slow or fast brushing.

**Figure 3:**
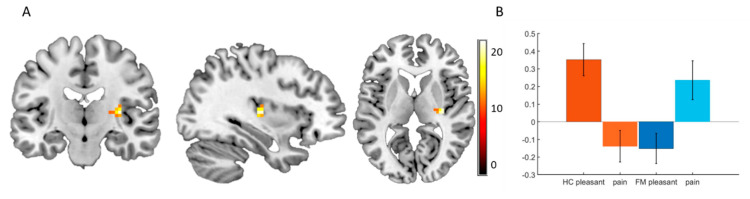
**Insula activity differs between FM patients and HC during rating period.** (**A**) Interaction group*ratings type (pleasantness and pain). During the rating period, we found a group*rating-type interaction in the posterior insula ([33–198], F = 22.67, Z = 4.39 p = 0.001 FWE, SVC for posterior insula), color scale depicts the F-values. (**B**) Beta values extracted from a 6 mm radius sphere around the peak of the interaction in the posterior insula [33–198].

Since we found a difference in ratings between HC and FM, we also explored group differences during the rating period. Activation in posterior insula was related to whether the subjects were rating on the pleasantness or the pain scale (Figure 3A). While HC showed activation in this area during pleasantness ratings and deactivation during pain ratings, patients showed the opposite pattern, i.e., deactivation during pleasantness ratings and activation during pain ratings (Figure 3B).

### 3.4. Voxel-Based Morphometry

**Figure 4:**
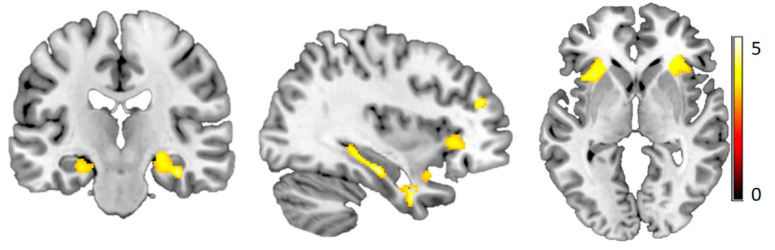
Reduced grey matter in FM. FM showed reduced grey matter density in bilateral insula and hippocampus ([35–19–1], *p* < 0.001, cluster size = 50), color scale depicts the *t*-values.

VBM analysis comparing HC and FM revealed reduced grey matter density in patients in the bilateral hippocampus and anterior insula. This difference was even stronger when including age as a covariate of no interest (Figure 4 and Table 2). There was no area where FM had a higher grey matter density than HC.

## 4. Discussion

In the current study, we found intact neural processing of positive affective touch in FM patients. However, slow soft brushing was reported as less pleasant by FM patients than healthy controls. We found that FM patients differed from HC during the rating of perceived pleasantness and pain. FM patients showed deactivation in the right posterior insula during the pleasantness rating and activation during the pain rating, the opposite pattern of what was observed in the HC.

### 4.1. Behavior

The pathophysiology of FM is not well-understood. Both peripheral and central nervous system alterations are involved in the development and maintenance of FM. Hence, alterations in the brain including neuroinflammation and activation of glial cells, nociception-driven amplification of neural signaling (central sensitization), opioidergic dysregulation, and impaired top-down modulation were found, as well as signs of systemic low-grade inflammation (e.g., regarding cytokine profile and inflammatory lipids) and nociceptor and muscle protein changes [28,29,30,31,32,33,34,35,36,37,38,39,40]. In an earlier psychophysical study in FM patients, we found that pleasantness ratings to brushing were normal, but the distinction between slow CT-optimal and fast CT-suboptimal brushing was diminished [8]. In the current study, we found that while the affective touch sensitivity (slow versus fast) was preserved, the pleasantness ratings were significantly lower in FM. Despite these differences which, in part at least, could be due to the heterogeneity of the condition [41], there is likely a disturbance in the affective touch system. To confirm whether this has a peripheral involvement would require electrophysiological recordings (microneurography) from CT afferents in FM patients.

The finding that HC did not rate the pleasantness of slow and fast brushing differently needs further consideration. Although average pleasantness ratings were descriptively higher for slow than for fast brushing, they were not statistically different. Previous studies have reported that people rate slow, CT-optimal brushing velocities as more pleasant than fast, CT-non-optimal brushing [3,26,42]. However, a recent study challenged the view of an inverted U-shaped curve for pleasantness ratings that peaks at the CT-optimal velocity, suggesting that the inverted U-shape can only be found as a group average [43]. Our sample might have contained healthy individuals who did not differentiate as much between a slow and fast touch. Another, possibly complementary explanation, might be a ceiling effect. This might be driven by a number of factors; the MRI-scanner environment was loud, uncomfortable, and boring, so any positive stimulus might be evaluated as especially pleasant. In addition, the combination with a question on pain experience might drive an overly positive evaluation of the brushing stimuli.

### 4.2. Functional Imaging

We found differential processing during the evaluative period for the two groups and conditions. While HC showed activation in posterior insula during pleasantness evaluation and deactivation during pain evaluation, FM showed deactivation during pleasantness evaluation and activation during pain evaluation. This result should be considered preliminary as it was the result of an exploratory analysis. That the cluster of the interaction effect did not directly overlap with the main effect of slow versus fast touch was not surprising, since the evaluative processing of the current experience was a different, hierarchically higher processing, and might therefore have involved different clusters within the insula [44,45,46,47,48].

FM patients display hyper-sensitivity to a range of sensory stimuli [49], so it has been suggested that this might be a general hypersensitivity syndrome [50]. Our results suggest that FM patients do not exhibit hypersensitivity to pleasant touch at an early processing stage, as we found no difference in neural activation in the posterior insula. However, we found altered activation patterns during the evaluation of positive stimuli and reporting of current pain levels, suggesting dysfunctional evaluative processes. This was in line with other studies; for instance, a study in which FM patients were compared to subjects with masochistic behavior found an alteration in the late response to tactile stimulation in the primary somatosensory cortex, as measured by magnetoencephalography [51]. The amplitude of this cortical response was inversely correlated with pain catastrophizing. Dysfunctional evaluative processing of a pleasant stimulus might be associated with anhedonia in FM. FM patients are less efficient at modulating their pain perception through concomitant positive stimuli [52], however, we found a small but significant reduction in pain ratings after slow brushing (rated as pleasant by the patients). Pain catastrophizing affects pain sensitivity (i.e., pain thresholds for pressure, cold, or heat) as reported in various cohorts of patients with chronic pain [53,54]. However, correlations are moderate, and the pain sensitivity is not solely explained by psychological aspects. Moreover, catastrophizing is related to fear of pain but also these correlations are moderate [55,56,57].

### 4.3. VBM

Alterations in grey matter in many brain regions related to pain processing have been reported in several studies (for review see [58]). These include grey matter increases in the cerebellum and the striatum [59] and decreases in the brainstem, anterior and posterior cingulate cortices, prefrontal cortex, parahippocampal gyrus, and hippocampus [60,61]. For certain areas, interaction with age was also reported, e.g., increased grey matter in the insula of patients younger than 50 years [62]. In the current study, we found decreased grey matter density in FM, in the bilateral hippocampus and insula—a reduction that was more pronounced when age was included (as a covariate of no interest). However, we found no brain region where FM patients had higher grey matter density than HC.

The hippocampal grey matter is decreased in people suffering from stress [63] and post-traumatic stress disorder [64]. This is consistent with a high prevalence of early life stress and adverse events in FM [65,66]. In our FM cohort, we found high distress, pain catastrophizing and insomnia, and a low perceived state of health. The insula plays an important role in the perception of one’s own body and sensations created within [48], specifically pain [67]. The anterior insula is involved in stratifying sensations into painful and non-painful [68]. The reduction in the anterior insular grey matter observed here might relate to alterations in the pain network in FM, as has been suggested previously [69].

### 4.4. Limitations and Future Direction

There are several limitations that need to be considered when interpreting our results. FM is a condition of highly varied symptoms and severity; therefore, patient samples tend to be heterogeneous. Larger and longitudinal studies are needed to distinguish subgroups within FM. Here, we focused on brushing for its affective attribute and link to C-tactile fibers, and questionnaires to capture the clinical characteristics, but we did not perform a detailed battery of quantitative somatosensory tests [70,71,72]; the combination of these could provide important insights into the peripheral nerve function, the role of top-down modulation, and the broader interplay between somatosensory and affective systems in aberrant pain states. To further disentangle perception and evaluation of sensory inputs, future studies could compare measures of hypersensitivity in the tactile domain with other sensory domains [73]. Physical activity is another important factor—while exercise leads to hypoalgesia in healthy individuals, it leads to hyperalgesia in patients with FM [74]. Further, FM patients have a heightened sensitivity to activity-related increases in pain [56]. Here, we simply asked patients to rate the extent to which pain affected their activities of daily living, leisure, and work. In future studies it would be interesting to explore this in detail using, for instance, the Sensitivity to Physical Activity (SPA) measures that are associated with clinical indices of pain hypersensitivity [75]. Another interesting future direction would be to focus on neurotransmitters such as glutamate, which is elevated in the insula in FM [11]. Glutamate could be tracked using biosensors [76] or could be measured in the brain using magnetic resonance spectroscopy [77], and these measures could then be related to behavioral and functional differences in FM patients.

Taken together, our results suggest intact early-stage sensory processing of positive tactile stimuli but dysfunctional evaluative processing. These findings contribute to our understanding of the mechanisms underlying anhedonia in FM.

## Figures and Tables

**Table 1:** Age, pain characteristics, psychological variables, the impact of pain and health aspects in the fibromyalgia group (FM) and in the healthy controls (HC); mean and one standard deviation (SD).

Group	HC	*n* = 29	FM	*n* = 31	Statistics
Variables	Mean	SD	Mean	SD	*p*-Value
Age (years)	42.7	10.1	39.2	11.4	0.219
Systolic BP (mm Hg)	113.2	8.8	121.5	12.9	0.006
Diastolic BP (mm Hg)	75.3	8.5	80.6	10.6	0.040
Number of tender points	0.3	0.9	16.7	1.5	<0.001
FM duration (years)			4.4	5.0	NA
Height (m)	1.69	0.06	1.66	0.06	0.090
Weight (kg)	68.4	10.9	81.4	19.2	0.002
BMI (kg/m^2^)	23.8	3.1	29.3	6.3	<0.001
Pain intensity current	0.0	0.0	5.7	1.8	<0.001
Pain intensity 4w	0.0	0.0	5.9	1.8	<0.001
HADS-Depression	1.4	1.7	6.0	3.6	<0.001
HADS-Anxiety	2.6	2.3	7.8	4.0	<0.001
PCS	11.8	9.2	20.3	10.5	0.001
ISI	4.5	4.5	13.4	5.9	<0.001
Pain hindrance ADL			5.7	2.3	NA
Pain hindrance leisure			5.7	2.5	NA
Pain hindrance work			4.9	2.7	NA
EQ-VAS	86.8	7.8	53.3	19.5	<0.001

BP = blood pressure; HADS = Hospital Anxiety and Depression Scale; PCS = Pain Catastrophizing Scale; ISI = Insomnia Severity Index; EQ-VAS = the health scale of EQ-5D (European Quality of Life instrument); w = weeks; NA = not applicable.

**Table 2:** Regions with reduced grey matter density in FM compared to HC. Age was included as covariate of no interest. *t*-test at the whole brain level, thresholded at *p* < 0.001 uncorrected.

Region	x	y	z	Hemi-Sphere	Cluster Size	*t*	*p*
Hippocampus	36	−22.5	−18	Right	469	4.98	>0.0001
Parahippocampal Gyrus	37.5	−31.5	−12	Right		4.68	>0.0001
	24	−19.5	−9			3.91	0.0001
	−24	−19.5	−16.5	Left	146	3.94	0.0002
Middle Frontal Gyrus	31.5	45	22.5	Right	233	4.69	>0.0001
Rectal Gyrus	3	37.5	−28.5	Right	339	4.51	>0.0001
Uncus	34.5	0	−34.5	Right	505	4.43	>0.0001
	−34.5	1.5	−28.5	Left	215	3.69	0.0003
Superior Temporal Gyrus	46.5	0	−16.5	Right		3.91	0.0001
	−40.5	6	−18	Left		3.49	0.0005
Subcallosal Gyrus	13.5	6	−19.5	Right	288	4.29	>0.0001
Anterior Cingulate	9	18	−12	Right		3.51	0.0004
Anterior Insula	−28.5	21	1.5	Left	764	4.20	>0.0001
	27	25.5	0	Right	430	4.13	0.0001
Middle Occipital Gyrus	−22.5	−88.5	4.5	Left	175	4.18	0.0001
Inferior Frontal Gyrus	37.5	24	−1.5	Right		3.77	0.0002
Cingulate Gyrus	−6	−10.5	43.5	Left	131	4.07	0.0001
	−9	−10.5	36			3.72	0.0001
Cerebellum	−22.5	−31.5	−33	Left	72	3.69	0.0003
